# Is pharmacogenetics being racialized? An investigation into the reinscription of racial beliefs in modern biomedicine

**DOI:** 10.1007/s11017-025-09723-4

**Published:** 2025-08-22

**Authors:** Lena Steimle, Christina Schües

**Affiliations:** 1 Faculty of Medicine, University of Lübeck, Ratzeburger Allee 160, 23562 Lübeck, Germany; 2 Institute for the History of Medicine and Science Studies, University of Lübeck, Königstraße 42, 23552 Lübeck, Germany

**Keywords:** Racialization, Pharmacogenetics, Genetic diversity, Racism

## Abstract

Racial classifications have a complex and troubled past in the social and scientific history of humankind. They are the result of racism and have been used to devalue and degrade non-White people. Although the concept of race has acquired a social component and the genetic similarity of people based on race has been proven, the category is increasingly used in pharmacogenetic studies to create biased study populations under the guise of personalized medicine. The heart failure drug BiDil, which was only approved for Black people in 2005, gained particular notoriety. Its race-specific approval reignited the debate about the assumption that there are genetic differences between people of different races and led to a further biopolitical instrumentalization of the term ‘race’. Despite the expiry of the race-specific patent, race-specific labeling continues to take place in research. In this paper, these racializations of pharmacogenetics are examined, explaining how they arise and how they are kept alive. Finally, this paper argues for better moderation of race in pharmacogenetic studies.

## Introduction

Hopes are high as pharmacogentic research into on human diversity increasingly detects potential disease genes that will lead to improved individualized therapies for said diseases in the future. However, many suspect that this research is racialized in discriminatory ways. Despite the fact that there is an emerging resistance to racial concepts (due to the fact that the genomes of two people are 99.9 percent identical when compared genetically), the concept of race remains at the forefront of much pharmacogenetic research and therapy, with racializing studies focusing on the 0.1 percent^1^ that can vary between people [1, 2]. While these genetic differences can determine both one’s appearance and one’s individual susceptibility to disease, most lack phenotypical impact [3]. Instead of interpreting these findings as confirmation of the non-existence of human races, racial differentiation studies continue to evolve.

In this article, we argue that the racializing that still occurs today is driven by the fear of alienation, the desire for segregation, and the maintenance of a white supremacist colonial narrative of the fear of mixing and degeneracy.

It is important to note some points for context. First, the concept of race has always been a fluid, ambiguous, and indeterminate term that has served as a category for various attributions. Critically, it has always been used in a pejorative and defamatory way, and therefore “the concept of race […] is the result of racism and not its precondition” [5].

Second, it must be acknowledged that polymorphisms are crucial for the detection of genetic diversity [6]. These are sequence variants of a gene within a population. Allele frequencies of the variant allele of > 1 percent in the population are referred to as polymorphisms. All frequencies < 1 percent are referred to as mutations. A polymorphism can, therefore, not be explained solely by the repeated occurrence of a mutation, but represents a stable variation and can, therefore, be inherited. There are different types of these sequence variations. At 90 percent, single nucleotide polymorphisms (SNPs) are the most common form of inter-individual genetic variability. A single SNP has relatively low information content; its strength lies in the frequency of its occurrence [7]. As SNPs are relatively stable, they are suitable for genetic studies. For example, if a base pair coding for a different amino acid is exchanged, this can change the activity of an enzyme and consequently also influence the phenotype of an individual. Within one study population, while 85 percent of all genetic differences were found, only 15 percent could be detected between populations [8, p. 287]. Of this 15 percent, about 10 percent of the total variation is accounted for by differences between populations living on different continents and only 5 percent by differences between populations on the same continent [9].

It can be assumed that the migration of people from Africa, but also migration throughout the world, has created the possibility that some SNPs are more common in some populations than in others [10]. Every human carries SNP variations that, based on different time intervals, refer to earlier ancestors and their original genetic diversity [11]. However, how populations are defined, how DNA is analyzed, and which populations are considered more homogeneous or heterogeneous all play a role.

In the following, we will firstly briefly recapitulate what it means to attribute the label of race to people. Secondly, we will discuss the racialization of disease, especially of heart failure. The main issue of the article will be, thirdly, the discussion about pharmacogenetics, as exemplified by the implementation of the drug BiDil and its consequences. We will then discuss its relevancy for today's practices and discussions about personalized medicine. Overall, our research aims to show a deep ambivalence with regard to the labeling of 'race'. The following question lies in the background of this discussion: Would a more strict labeling of race avoid racism? We think that not; but it is at least ambiguous. While the using the criteria of race may lead to discrimination (i.e. racism), it could also be supportive for research or therapy.

## The ambiguity of race-labels

Despite the modern social component of the term, race is attributed depending on (presumed) ancestry and phenotypic appearance, such as facial features, body shape, and, above all, skin color [12, 13]. The current statistics on a person's race in the USA are collected by the *US Office of Management and Budget* (OMB). The OMB standards (No. 15) specify five possible racial categories: American Indian/Alaska Native, Asian, Black/African American, Native Hawaiian/Other Pacific Islander and White. The assignment is based on self-identification, and since 1997, it has been possible to assign oneself to more than one race [14, 15].

A person's race is additionally recorded by the National Center for Health Statistics (NCHS) based on information on the birth or death certificate. Here, race is obviously attributed from the outside. This can lead to considerable differences in attribution and misclassifications [16]. Therefore, it is theoretically possible to be assigned to three different races at birth, during a census, and at death [17]. Depending on the statistics considered (OMB or NCHS), different numbers of people with different *racial* affiliations live in the US at different times. This makes the data highly variable and consequently reduces its significance. In addition, the attribution system in the US has changed over the years. At time of birth, the race of a newborn is usually inferred from the race of the parents. Until 1989, newborns only counted as White if both parents were White. Historically, this is derived from the one-drop-rule^2^ [17, p. 467]. Since 1989, only the race of the mother determined the race of the child. This amendment altered the statistics, and the number of births of White children increased [17]. This shows how arbitrarily race is linked to skin color and how inaccurate and unreliable the data collection is [17, p. 267].

It is not only the concept of race that is ambiguous, but also the selected categories of race. The description of one's own identity (e.g. as an African American) can be used to express social and political identity [18]. However, this self-designation is not synonymous with (exclusive) African descent for all African Americans. Self-defining Black people and self-defining White people have African and European ancestry. Since race is still strongly influenced by phenotypic appearance, regardless of whether it is self-attributed or attributed by others, the implicit information on ancestry that is supposed to be collected in genetic studies is often too imprecise or simply wrong. According to the geneticist Mersha and the molecular biologist Abebe, genetic analyses show that people who describe themselves as African American are sometimes up to 99 percent of European descent. In contrast, people who describe themselves as European have a significant admixture of African descent [19]. African American is an imprecise category for detecting genetic differences, as people with ancestry from East, West, South, and North Africa can have significant genetic differences in addition to various proportions of European ancestry [20]. African Americans are genetically and socio-politically a very heterogeneous group and race is therefore a poor value to indicate a consistent genetic background for them.

The self-attribution of race is most commonly used for data collection in biomedical research. However, these self-attributions are not immutable and permanent. On average, 5–12 percent of all Americans change their self-perception of their race over time [21]. According to sociological-empirical studies, people whose parents belong to two different OMB races are particularly likely to change their self-identified race [22, 23]. The phenomenon is emblematic of the interaction between racial fluidity and racial inequality and therefore primarily affects minorities and multiracial populations [16, 24]. Americans who originally identified as non-White change their race by an average of 20 percent [21]. However, changing self-attributions within a lifetime makes censuses and surveys inconsistent. Due to the mixing of intrinsic and extrinsic forms of attribution and the change in categorization over time, the statistics collected are very limited. This illustrates how difficult it is to make self-attributions statistically explicit when it comes to recording genetic differences. Race can therefore not be understood as a “consistent identity but as a number of conflicting dimensions” [25, p. 1310].

This process, which is labeled as ‘racialization,’ refers to an act of ascribing oneself or being ascribed to a particular race. Racialization generates or reproduces knowledge of supposed categories that are based on the attribution system of the construct of race. Racialization means a scientific classification and hierarchization of people of different skin colors, cultures, or languages. It is a form of stigmatization and stereotyping in which people have ascribed characteristics that make them representative of their race due to their supposed group membership. BiPoC (i.e. Black, Indigenous, people of color) are subject to racialization, as White people perceive themselves as normative and raceless. Racialized people are often defined as different and as abnormal [26]. Just like the concept of race, besides its racist implications, racialization is a fluid process that makes it nearly impossible to use it as a basis for statistics in biomedical studies.^3^

## The racialization of genetic traits on skin color

African Americans are often considered members of a mixed population because they were primarily considered for collecting SNPs in admixture mapping studies [27]. Native Americans, however, are perceived as isolated and inbred populations, although intercultural, interlinguistic and interethnic marriage and reproduction have occurred just as often there as in any other region labeled as a population. This is due to biased categorizations that were consciously decided upon and not due to shared genomic structures [27]. Viewing Native Americans as genetically and culturally isolated disregards the constant mixing between populations; populations labeled as isolated today were not necessarily isolated in the past [28, p. 81].

Although African Americans are perceived as a rather heterogeneous group through admixture mapping, they are often viewed as a highly simplified homogeneous group in disease and medication distribution studies, even though “DNA analysis of present-day Africans reveals fourteen ancestral population clusters” [29, p. 59]. Their genetics are the most variable compared to other populations. It therefore seems absurd that African Americans in particular are attributed a certain collection of alleles that make them more prone to diseases. “Even the term African American makes no sense genetically, as it implies a unified population. The 20 percent average admixture of European alleles in African Americans, as well as the great genetic variation among different American populations, belies this biogeographic generalization” [29, p. 59]. The possibility of assigning continental variants is seen as a valid reflection of biological differences between races [27]. Biological differences between people can lead to different disease risks. However, these multifactorial characteristics do not follow racial categories. The idea that such genetic categories exist leads to an increase in stereotyping and racialization.

Skin color, a frequently racialized characteristic, has long been used to categorize and devalue people deviating from the white norm, often resulting in discrimination against BiPoC [30]. In the USA, the genetically diverse Black population is often homogenized in medical studies based on skin pigmentation [31]. Skin color is a multifactorial trait influenced by various genes and environmental factors [6, p. 778]. Increased melanin production, which protects against UV-induced folic acid degradation, correlates with high UV radiation at the equator [32, 33]. This evolutionary adaptation means darker skin offers a selection advantage in sunny regions, while lighter skin is advantageous in areas with less sun, aiding vitamin D synthesis. Despite similar pigmentation, populations can be genetically diverse, indicating convergent evolution with different mutations leading to similar phenotypes [34–36].

The highest genetic variation is generally found in people from Africa [6, p. 921; 37, p. 1172; 38, p. 867; 39, p. 294]. This speaks in favor of the theory of emigration from Africa, as humanity had the longest time to develop genetic diversity there. Due to emigration from Africa, people on other continents have relatively less genetic diversity [10, p. 601]. Not enough time has passed for the emergence of further genetic variants that are not merely superficial, such as skin color or hair texture [40]. However, as these traits are particularly striking, we are tempted to suspect further differences [40, 41].

Overall, the close relationship of humans within the species compared to other species is striking. Humans have the lowest species diversity among primates [8, p. 287]. In other words, each human population contains most of the genetic variability of the entire species [42]. There are different allele frequencies within human populations, but these are more gradual than significantly different. Some alleles are rare in some populations and common in other populations. However, there is no allele that occurs exclusively within a single population as an allele is not fixed within a population [39, p. 288; [43, 44]. The different pigmentation of the skin only provides information about the environmental conditions in which people lived at their place of origin, which leads to adaptive responses in different environments. Therefore, no conclusions can be drawn about supposed racial categories or a specific region based on skin [35]. Such categories are not only inappropriate, but simply cannot be defined. A consideration of the example of heart disease shows well how racializations of genetic traits have constantly been introduced.

## The racialization of diseases: heart failure

For around 20 years, ischemic heart disease has been the most common cause of death worldwide [45]. Particularly in industrialized nations, heart failure is on the rise due to increasing age. Heart failure happens when the heart cannot maintain the necessary pumping capacity to supply the body. It can occur due to pre-existing conditions such as long-term high blood pressure, coronary heart disease, valvular heart disease, and myocarditis [46]. As a complex secondary disease, heart failure is highly polygenic. Although the severity of the risk of the disease may depend on various genes that have been identified, it is difficult to determine to what extent they contribute to the risk and whether they contribute at all [47].

Heart failure can be considered a racialized disease because the causes, treatment options, and severity of the disease are being researched, particularly in the United States, in a way that shows and highlights clear differences between White and Black populations [48].

The mortality rate of Black patients with heart failure is higher than that of White patients in the USA [10]. There may be socially determined reason for this, such as poor access to cardiovascular care, poorer adherence to guideline-compliant pharmacological therapy, and/or reduced referral to further therapies [49]. Black patients are less likely to receive medication or concomitant device therapy for their heart failure and are less likely to be seen by cardiologists [50].

One of the strongest medical risk factors for heart failure is permanently high blood pressure. The permanently high pressure causes the heart muscle to thicken and become inelastic. Due to the increasing stiffness, the ventricle no longer fills sufficiently, which is why the heart's pumping capacity decreases. Good control of high blood pressure prevents the subsequent occurrence of heart failure. High blood pressure is likewise more common in African Americans [51]. As it is a polygenetic disease, genes that regulate the renin–angiotensin–aldosterone system (RAAS) may be affected. The RAAS is significantly involved in the increase in blood pressure and, as a pharmacological site of action, can be decisive for the adaptation of therapy. Angiotensin-converting enzyme inhibitors (ACE inhibitors) are important for blood pressure therapy and for the basic therapy of heart failure. Renin deficiency is relatively common in African Americans. It is therefore assumed that ACE inhibitors, because they additionally suppress the RAAS system, are less effective in African Americans than in patients of European descent [52].

However, according to a meta-analysis by nephrologist Ashwini Sehgal, few differences were found in the response to antihypertensive drugs between White and Black people. A meta-analysis of 15 clinical trials (1983–2003) found that, on average, 80–95 percent of all Black and White subjects included responded similarly to common antihypertensive medications [53]. A possible explanation for the discrepancy in reaching target blood pressure with ACE inhibitors, which was achieved in more white subjects, could be the 4 mmHg higher baseline diastolic blood pressure values of the Black subjects [53]. Baseline blood pressure levels were decisive for the detected racial difference. There was only an 8 percent racial difference in beta-blocker^4^ use when both Black and White subjects were 6 mmHg above target blood pressure. The racial differences widened to 30 percent when Black subjects were 10 mmHg above the treatment target at baseline. These crucial influences of baseline blood pressure on the efficacy of a drug when looking at target blood pressure could overestimate detected racial differences in trials of antihypertensive drugs. Results may be misinterpreted if baseline blood pressure values are not reported separately for White and Black subjects, but target blood pressure values are [53]. According to physicians Lizzy Brewster and Yackoob Seedat, lower renin levels were more common in people of African descent but did not predict or poorly predict response to antihypertensive medications [52]. Consequently, race is a poor predictor of response to antihypertensive medication. Black and White subjects overlap too much in their response to these drugs. Instead of using race as a predictor, efficacy in individual patients, indication, and cost of the drug should be included [53]. Cost can play a role in the decision for or against a medication, as long-term care can only be secured if patients are able to pay for and take the medication. Accordingly, each patient must be considered individually regarding comorbidities. For example, the use of ACE inhibitors is beneficial for patients with chronic kidney disease, regardless of their ancestry, as they have renal and cardioprotective effects [53, 54]. Indeed, “no known biomarker, pharmacogenomic, or pharmacokinetic profile consistently predicts therapeutic responses […] in African Americans” [54, pp. 4–5]. In most cases, patients’ arterial hypertension requires a combination therapy of medications that are considered equally effective in all races. In general, individualized therapy should be established based on a comprehensive examination that includes family history, clinical characteristics, lifestyle, assessment of end organ damage, and careful follow-up [54].

Structural racism itself may be an indirect cause of hypertension in Black people. In the Cardiovascular Risk Development in Young Adults (CARDIA) study, self-perceived racial discrimination was positively correlated with blood pressure [55]. Systematic racism that results in unequal treatment and discrimination against BiPoC in areas such as housing, benefits, health care, wages, and employment can increase stress levels. This increased stress level leads to higher blood pressure levels. This is one suggestion of how racism can have a direct impact on the burden of heart disease in Black people.

The racialization of heart failure results from different, supposedly race-based assessments of the severity of the disease. In medicine, racial classifications are used as diagnostic algorithms and practice guidelines. These algorithms are intended to assist medical personnel in making an individualized clinical decision for or with the patient. Race-based correction factors are used to propagate and reproduce racial disparities [56]. In the previous American Heart Association (AHA) Get with the Guidelines-Heart Failure Risk Score, patients identified as non-Black were assigned three additional points for risk of death on hospital admission [57]. This means that Black patients were associated with a lower risk of death at hospital admission based solely on their skin color and perceived race, and were less likely to be referred to a cardiologist as a result [56]. In this context, being Black equates with a lower need for further referral to a specialist. As studies have confirmed, Black patients with heart failure repeatedly face the risk of being treated worse than White patients [58, 59]. Although the AHA guideline for the management of heart failure published in 2022 emphasizes that Black people are more likely to develop heart failure, “these differences are driven mostly by social circumstances; a biological premise or genetic explanation for diseases or disease severity should not be inferred by race” [60, p. e973]. A study on Nigerians revealed that they have the lowest rates of arterial hypertension in the world. They have lower rates than the White population in Germany [61]. Overall, the hypertension rates of Black people are not exceptionally high in international comparison [62]. This makes it apparent that the poorer healthcare of Black people in the US and in Europe is the cause of the increased burden of disease and not another genetic condition [63]. To better understand the attribution of race, the role of pharmacogenetics, and the upshoot for medical research, we will now examine their mutual entanglement.

## The relationship between race, pharmacogenetics, and medical research

Pharmacogenetics has developed as a branch of pharmacology to study the relationship between genetics and drug response. It is still a young discipline that aims to treat people individually depending on their gender, age, environmental factors, and personal genetic make-up [6, p. 797]. By taking these factors into account, it should be possible to tailor suitable medications and reduce adverse drug reactions, thereby increasing patient adherence to treatment [64]. Pharmacogenetics investigates genetic differences between people, as these differences can lead to different reactions to the active substance(s) from person to person. Interindividual differences in drug metabolism are mainly determined by the cytochrome P450 (CYP) system [65]. These heme proteins have specific enzymatic activity crucial for the metabolism of many drugs. CYP enzyme induction can lead to a faster and CYP inhibition to a slower conversion of the substrates. Depending on whether the active substance is degraded or activated via CYP, the effect can be weakened or intensified, which can lead to adverse drug reactions. Due to the complex and not yet fully understood genetics and inheritance of common polygenetic diseases such as cardiovascular diseases, as well as their strong dependence on lifestyle and environmental factors, it is currently not possible to determine an individual genetic risk for these diseases [66]. In this vein it is not yet possible to offer patients an individual therapy for every disease according to their genome.

To avoid having to examine each genome individually, categories are formed using surrogate parameters, which are supposed to enable meaningful predictions in summarized groups. At first glance, this may seem to contradict the individualized approach of personalized medicine, but it makes research less complex and, above all, more economical. Critically discussed categories such as race are used in pharmacogenetic research as surrogate parameters to detect possible aberrant drug reactions or an increased genetic risk of disease. Accordingly, the use of race categorization has steadily increased in studies and journal articles in recent years; publication guidelines have contributed to the racialization of research and its results.

*The New England Journal of Medicine* (NEJM) has decided to no longer publish studies that do not include information on the race of subjects. This can in no way be directly understood as racialization. According to the NEJM, background information on subjects such as gender, age, race or ethnic group and origin is intended to make underrepresented groups such as ethnic minorities more visible and to make the selection of study participants more diverse [67]. This demonstrates that it is often not a personal decision by researchers and institutions that lead to the inclusion of race [44, 61]. Important to the establishment of these research criteria were various federal mandates requiring the inclusion of women and minorities in clinical investigations [68]. Two guidelines issued by the US Food and Drug Administration (FDA) in 1999 and 2005 recommend the inclusion of race and ethnicity in pharmacological studies if they are designed or intended to lead to the approval of new drugs [61, p. 26].

In medical research, the OMB standards are mainly used to record race, which is collected by self-report. These categories, which were created for bureaucratic and political purposes, were never intended to serve as scientific variables [69]. Therefore, these are unreliable approximations that do not capture the complexity of patients' racial affiliations [56, p. 879]. In particular, the rather technical, casual, and supposedly neutral categorization of OMB standards lends an “aura of legitimacy” to the link between race and genetics [61, p. 34]. However, self-categorizations change depending on context and current political definitions. Depending on the indication of the question, respondents make different statements about their own identity in different surveys and at different times. Longitudinal studies have shown that the attribution of race has proven to be inconsistent over time [25]. It is noticeable that there is usually a lack of definition of race and/or reason for its use as a category in biomedical studies. In a study by doctors Ma et al., a total of 1.152 biomedical journals such as the *Lancet* and *NEJM* from the years 1990–2003 were examined [70]. In only 16 percent of the research articles were the reasons for collecting information on race explicitly stated. In more than half of the selected articles, no information was provided on the socioeconomic status of the study participants, which suggests race was viewed as a purely biological category [70]. It is often not recorded why this variable is even necessary in the context of the research. Moreover, different and usually imprecise meanings are attached to the concept of race [61, p. 40].

An interview study of 30 geneticists who used OMB races as categories in their studies revealed they were aware of the arbitrariness of the classification and its scientific inadequacy [69]. They defended their work by saying that they were only using race as a temporary substitute parameter before better categories were available: “None had developed overt strategies for addressing these inadequacies, with many instead asserting that science will inevitably correct itself and saying that meanwhile researchers should ‘be careful’ in the language chosen for reporting findings.” [69, p. 1]. Accordingly, the reasons for the careless processing of categorization may be global, structural, and often unquestioned (power) structures that use race in problematic ways [61, p. 25]. Thus, “geneticists seem to be convinced of the nonexistence of race yet need social categories of difference to order their data and to interpret the results” [2, p. 439]. But this can lead to the assumption that a biological explanation is behind the unequal outcomes for White people and BiPoC. Therefore, definitions and explanations of why such categories are used are essential to avoid inadvertently reinforcing structural racism.

In pharmacological and pharmacogenetic research, the White (average) man is the standard subject: “Whites as the privileged group take their identity as the norm or standard by which other groups are measured” [71, p. 125]. However, most drugs are not specifically declared as drugs for Whites only or as race-based drugs for Whites, as “drugs tested on Whites are [considered] applicable for all humans” [72, p. 22]. In contrast, drugs tested on Black people are not automatically considered universally applicable drugs for all people. Black people are therefore considered less representative of humanity than White people in pharmacogenetic research.

Why is the use of race as a category attractive for pharmacogenetic research? It implies some degree of biological relatedness, albeit an imprecise and biased one, as information on pharmacogenetic response to drugs in genetically related individuals is scarce [73, p. 233]. Pharmacogenetic studies have shown that genetic polymorphisms relevant to drug effects do not necessarily correlate with race. In a study by the Institute for Pharmacogenomics and Individualized Therapy at the University of North Carolina, which investigated whether race can be used to predict drug-related genetic variants, researchers found that most genetic variation is determined by individual variation and not by race [74]. Race is therefore not a good surrogate for individualized therapies [19, 74]. The issues with race as a category and as self-attribution shall now be examined with reference to BiDil, or ‘the Black pill.’

## BiDil: a personalized drug?

The drug BiDil was approved by the Food and Drug Administration (FDA) in the USA in 2005 for the treatment of heart failure. It was the first race-based drug to be approved only for patients who self-identify as Black and suffer from heart failure. BiDil is a combination of the active components’ isosorbide dinitrate and hydralazine, which have already been used generically for the treatment of heart failure for many years [75–77]. Both agents (Bi) act by vasodilation (Dil) and thus lead to a reduction in oxygen consumption by the heart muscle cells. To analyze the development of BiDil, it is necessary to look at the V-HeFT I/ II and A-HeFT studies and the original publications that led to approval.

The results of V-HeFT I showed that although the combination of hydralazine and ISDN (H/I) led to a reduction in mortality, this difference was only of marginal statistical significance [78]. In the subsequent Vasodilator Heart Failure Trial II (V-HeFT II), the efficacy of the H/I combination was compared with enalapril, an angiotensin-converting enzyme inhibitor (ACE inhibitor). The mortality rate after two years was significantly lower in the enalapril group (18 percent) than in the H/I group (25 percent) [79]. The results of this study confirmed the positive effect of ACE inhibitors on mortality in the initial treatment of heart failure and made them the standard therapy [61, p. 54; 77].

BiDil’s application for approval was subsequently rejected by the FDA due to numerous problems with the study and the analysis [61, p. 56; 77]. Since the V-HeFT studies were not designed as registration studies, the endpoint criteria were not precise enough to interpret with sufficient certainty [61, p. 57; 78, 79, 80]. In addition, the V-HeFT I and II trials were terminated prematurely due to funding issues and therefore insufficient statistical data [1]. However, therapy with H/I seemed promising and approval of BiDil in the general population for the treatment of heart failure was considered possible. When both therapies were combined in patients with heart failure in whom ACE inhibitor therapy was not sufficient, the addition of H/I appeared to have a positive effect on mortality and quality of life. BiDil could be approved if followed by a properly controlled follow-up study with significant results [1, 77].

To gain BiDil's approval, Cohn, who himself emphasized that he had based V-HeFT I on a study designed twenty years earlier, reexamined the V-HeFT data with other researchers. Up to this point, none of the numerous articles that had been published on the V-HeFT studies had mentioned anything about a perceived race-specific difference in response to the H/I combination [61, p. 58]. Although 21 other non-race-specific traits were identified as targets for further analysis, it was decided to retrospectively examine the V-HeFT studies for the criterion of race [81]. This is possibly because of the favorable and uncomplicated possibility of using race by self-identification in a new study. In addition, retrospective categorization by race occurred at a time when US health policy was paying more attention to addressing racial and gender disparities.

The key discovery of the subsequently published results was that the annual mortality rate of Black patients who received H/I therapy as part of V-HeFT I was significantly lower than that of White subjects [82]. In the placebo group, there were no differences in the mortality rate [83]. However, the researchers pointed out that the data used for this conclusion came from “underpowered subset analyses and therefore must be viewed as hypothesis generating” [82, p. 186]. Nonetheless, the results would indicate that heart failure therapy “may be racially tailored” [82, p. 186]. Even though race has no real biological basis, they argued that a group of people who describe themselves as Black “may have common characteristics that are generally different from patients of other ethnicities” [84, pp. 128–135].

Finally, Cohn received approval from the FDA to conduct a new race-specific study for the potential approval of BiDil. Normally, drug approvals in the US are only granted if two large, well-controlled clinical trials are conducted prior to approval. However, the FDA allowed V-HeFT I and II to be counted as one large study. Strictly speaking, the result that BiDil could work better in African Americans was only achieved by the V-HeFT I study. In this study, only 49 African Americans received the active ingredients H/I [1, 85]. In the same year, the randomized, placebo-controlled, double-blind African American Heart Failure Trial (A-HeFT) began with 1050 male and female subjects who self-identified as Black. This is a rather small study population for a drug trial, but since it was assumed that this would be a kind of personalized medicine, this number was considered sufficient [86]. Subjects received either a placebo or BiDil in addition to their standard therapies [87]. Instead of comparing BiDil with another standard treatment for A-HeFT, the aim was to test whether BiDil could provide an additional, significant benefit to the standard treatment already received [84]. In July 2004, the study was terminated prematurely as a 43 percent lower mortality rate was found in the BiDil group [87–89]. A significantly improved composite outcome score based on mortality, hospitalization, and quality of life was found in the BiDil group.

On June 23, 2005, BiDil was approved by the FDA with a race-specific indication. This makes BiDil the first and so far, only race-specific drug ever approved [77]. In September 2009, NitroMed attempted to obtain additional patent protection and filed the patent application “Compositions for Treating Vascular Diseases Characterized by Nitric Oxide Insufficiency” [90]. This application was for a delayed-release version of BiDil to reduce dosing from three times daily to once daily. A specific racial effect was not mentioned in this application. When the category of race was no longer needed for patent protection, it was discarded in favor of finding a larger, non-racialized market [61, p. 66].

A study led by cardiology professor Karl Hammermeister concluded that BiDil was not associated with a significant reduction in mortality or hospitalizations in the more than 76.000 people included in his study [91]. In his study, different racial groups were formed for this purpose, but they did not differ in their results. This study became widely known in 2017 when a Republican congressman for Donald Trump tried to have it removed from a government website after having previously received campaign contributions from the BiDil manufacturing company Arbor Pharmaceuticals [92, pp. 265–266]. The race-specific patent expired in 2020.

The FDA viewed the approval of BiDil as another promising step towards personalized medicine [93]. In retrospect, however, BiDil cannot be considered a pharmacogenetically active drug because no specific genetic markers for its effect have been identified [86]. Similarly, there are no genetic markers that manifest themselves exclusively within a racially categorized group [89]. It has been asked: “Does the drug work better in dark-skinned blacks than in light-skinned blacks, in vanilla-colored versus caramel-colored, in deep chocolate-colored versus high yellow? What percentage of black blood, DNA, or skin color gradation must African Americans have to be good candidates for the drug and for it to work effectively?” [94, p. A9, 2004; 95, p. 172]. Accordingly, the continued use of race as a marker for underlying genetic differences could lead to many people continuing to be treated with drugs that do not work well for them [96]. Racial profiling in medicine cannot rule out serious errors of judgment if a person does not fit the stereotype that applies to his or her group. As Steven Epstein has said: “Even when we leave aside all the troublesome questions about how racial or ethnic group membership is defined in the first place—How do we handle those who are biracial or multiracial? […] can we really be certain that we will do the patient a favor by treating him or her as a representative member of the group?” [44, p. 219].

Keith Ferdinand, a cardiologist of the Association of Black Cardiologists, hypothesized that the effect of BiDil is related to many factors, such as blood pressure, socioeconomic status, and the stress it causes [97]. Despite the fact that race has no clear biological markers, he argued that the category could be important in studies to develop life-saving drugs [61, p. 51; 98]. According to Ferdinand and colleagues: “This drug has been shown to be beneficial when added to conventional modern therapy in patients who have moderate to severe heart failure and it was based on a study of 1,050 self-identified African Americans – period” [97].

It may have been wrongly assumed that small effects in different racial groups were due to sociocultural differences and large effects were due to biological differences [88]. Thus, the need for a retrospective race-based analysis of the V-HeFT studies was justified by the argument that African Americans were twice as likely to die of heart failure as Whites and would therefore require more specific therapy. Such a significant difference could not be explained by sociocultural factors alone and must therefore be related to genetic differences [61, 88, 99]. However, this data refers to 1981 and only denotes people under the age of 75. Data from 1995 for people over 65 showed that the ratio was 1.1:1 [88, 100]. This data was not included in A-HeFT, even though 94 percent of all heart failure deaths occurred in people over 65 [100]. Even more recent data from the National Center for Health Statistics, dating back to 1999, confirm the age-adjusted ratio of heart failure deaths in Black to White patients of 1.1:1 [61, p. 73]. However, in the new analysis of the data, the focus was on patients aged 45–64 years. This made the difference in mortality between Black and White subjects even greater. It is true that deaths due to heart failure are more common at a younger age in the Black population than in the White population. Why this is the case has not been clarified by the BiDil studies, but it is likely that sociocultural factors play a role. According to the article *Illuminating BiDil*, which was published in the journal *Nature* in 2005, “BiDil is more effective in blacks merely because the black population studied is more prone to die of heart disease at a younger age” [100, p. 903]. For this reason, research into the environmental and social factors that influence heart failure should be continued.

Critics of BiDil see race-specific approval as evidence of commercial and market incentives following previous rejection in the general population [101–103]. Only about 43 million dollars were spent on the A-HeFT study. By comparison, the average cost of bringing a new drug to market is estimated to be between 100 and 900 million dollars [104, 105]. The customized therapeutic approach of the A-HeFT study appears to have been an attractive model for savings [86, p. 745; 106].

Professors of epidemiology and biostatistics Ellison et al. demanded a subsequent combined estimate of the mortality rate of White and Black participants in V-HeFT I with those in V-HeFT II. This may have been intentionally omitted because it would have attenuated the magnitude of the racial differences found in V-HeFT I and would not have resulted in approval as a race-specific drug [102]. In addition, neither in V-HeFT I and II nor in comparison to A-HeFT were the formulations of the active ingredients H/I of the study drug bioequivalent [102]. In the case of hydralazine, the tablet formulation used in V-HeFT II showed significantly lower blood concentrations than the capsule formulation tested in V-HeFT I. The fixed-dose combination of BiDil as a single tablet resulted in different blood concentrations. According to Tam et al., “The apparently modest effect on survival observed in V-HeFT II could be explained in part by the poor bioavailability of hydralazine in the tablet form used in this study” [107]. Moreover, the different dosage forms as capsules or tablets can make a difference. It is unclear whether different results could have been achieved if the bioavailability of hydralazine had been the same in all studies. At the very least, this fact should have been checked before the start of the A-HeFT study, as the basic therapy for heart failure was as well different in the previous studies [102].

A study examining the benefits of H/I therapy in patients with decompensated heart failure in non-African American patients concluded that the addition of H/I “is associated with a more favorable hemodynamic profile and long-term clinical outcomes […] regardless of race” [108, p. 1]. Today, the Heart Failure Society of America recommends that patients who remain symptomatic despite optimized standard therapy should be treated with hydralazine and isosorbide dinitrate regardless of race [77].

Proponents of BiDil refer to race as a placeholder that is only used until better markers are found and cost-effective genetic screening is possible. However, we argue that race is a harmful and inaccurate marker that should have no place in pharmacological drug testing. Because when race is used as a variable in research, there is a tendency to understand the results obtained as a biological manifestation of racial differences and to assume genetic differences as the reason for different outcomes in terms of disease incidence and/or severity [109]. However, as these assumptions are rarely justified by a genetic characteristic, they may constitute a subtle form of racism [109]. This transforms health inequalities caused by social and economic inequalities into health inequalities that are perpetuated by biology and genetics. It is easier to label complicity in these disparities as genetic determinism than to understand them as the result of scientific racism and years of health discrimination.

## The relevancy of an ‘old’ story for today

It has been almost twenty years since BiDil became the first drug approved for Black patients only, and yet the discussion of racialization in pharmacogenetics is more relevant than ever. Since BiDil, there has been no further race-specific approval of a drug, yet *race*-specific drug prescriptions exist [110, 111]. Although numerous individual and specific factors are now known that enable personalized medicine, people are still categorized by race in pharmacogenetic studies. It is intended to serve as a rough guide for assessing genetic risk for disease and to enable evidence-based treatment recommendations [112]. The unquestioned further use of racialized data leads to a progressive racialization process. This makes a clear, interdisciplinary consensus on the appropriate use of data used to describe and categorize people even more important. In the words of Anne Pollock: “As novel as its FDA indication was, this drug represents just one moment in a long-standing and dynamic interplay of race, pharmaceuticals, and heart disease in America” [48, p. 1].

In the context of pharmacogenetics, it is important to classify people according to their individual characteristics to identify and explore genetic risks and treatment options. Non-genetic descriptors such as smoking habits, education, place of residence, physical activity, eating habits and experienced discrimination interact with genetic risk factors for disease [16, 19]. In gene-by-environment interaction research, non-genetic descriptors are all those factors that can influence a person's phenotype or health status outside of DNA. Therefore, in pharmacogenetic studies, it is essential to measure these environmental factors directly so that unexplained variance is not attributed to racial differences [68, p. 106]. Capturing genetic variation with comprehensible and, above all, comparable categories is challenging and complex. The excessive cost of individual genomic analysis is a recurring factor in justifying the use of distinguishing categories such as race. Although the cost of genome sequencing is decreasing, the cost of personalized medicines is increasing as they may only be available to a small target population [113]. Importantly, there are already examples of individual genetic characteristic screening, such as the HIV treatment drug abacavir, where individuals are tested for the presence of a risk allele associated with serious adverse drug reactions, regardless of their suspected genetic origin [114, p. 626]. Universal screening to detect genetic variations in a person's genome would eliminate the need for overarching racial categories or other broad umbrella terms and contribute to more specific, individualized, and safer therapy.

The very frequently used categories such as Black, Asian, or White alone show how phenotypic and geographical characteristics are mixed up in the recording of race. Individuals are not always comparable to the population to which they feel they belong or in which they were recorded [10, 73, 115–117]. The genetic variation collected by the racial category for a particular population and associated with an undesirable trait such as disease risk is transferred to an individual who may not possess that genetic trait [64, 116, 118]. At the same time, individuals are seen as representative of a large, diverse group. Non-White people are seen as exclusively representative of the racial group ascribed to them, while White people are seen as representative of humanity in general. The paradox of race-specific attributions is that the groups that are generally racialized are the least subject of current research. Genetic research continues to be conducted primarily on White people of European descent [119]. The underrepresentation of BiPoC in clinical trials further limits the assessability of recommendations for drug effects [120]. This carries the risk of people being prescribed medication that may not suit them. At the same time, there is a risk that people will be denied a drug that is effective for them because their racial group has not been tested for it or because there are recommendations not to use the drug.

Another important aspect is that differences in disease prevalence are often attributed to differences in genetic predisposition. Environmental and social causes that affect the disease burden and are relevant to the occurrence of different disease prevalence are sometimes not considered [121]. The use of racial terms to describe epidemiologic data perpetuates the notion that race per se places patients at differential risk of disease. This notion may cause race-based diagnostic bias [20, p. 873]. It is presented as an essential biological causal mechanism, and differences in disease prevalence are attributed to innate racial differences [20, p. 873].

According to geneticist Sarah Tishkoff, a patient's origin can play a role in the diagnosis and treatment of the disease if the physician is aware that the assessment of genetic variation within a population is limited and does not follow national boundaries or race [122]. Although statistical analyses have shown that differences between racial groups have been identified (i.e., there is such a thing as people of different ‘races’), these do not translate into good predictability of drug response at the individual level [111, 123]. Ultimately, as any physician would say, the individual must be treated and not the supposed population behind them. Pharmacogenomics must not replace clinical assessment. To truly optimize the use of personalized medicines, more meaningful and detailed categories than race must be used [124]. A person's geographic origin can provide limited information about a person's genetic background, but it is much more important and detailed when genome-based biomarkers of genetic similarity between people are examined.

There are now increasing efforts to better manage the use of race in medical research [68, 125, 126]. The editors of various journals call for a differentiated view of the category of race and, if it does not serve research discrimination, to avoid it. *The National Academies of Sciences, Engineering, and Medicine* (NASEM), an organization established by Congress and funded by the NIH, published recommendations in 2023 on how to better moderate population descriptors [68]. *The Journal of the American Medical Association* stipulates in its guidelines that studies to be published must explain the source of the determination of a person's race or ethnicity (e.g., whether race determination was made possible by self-report, by choice or by external attribution). In addition, information about the reason for and definition of the categorizations must be collected. Whether this decision was made by the researchers themselves or by a superordinate funding body must also be critically questioned. These groups understand that blindly adopting racial categories and definitions can lead to false conclusions about what is known about race. Authors are forced to think for themselves about the definition of the categories they use. This leads to an obligatory engagement of authors with the discourse on race. The deliberate decision to select and use population descriptors supports this debate [68, p. 43]. The study designs themselves should be critically scrutinized, especially regarding the actual significance of their results. The study design should therefore make a fundamental distinction between factors with biological and those with sociocultural significance and impact. A distinction should be made as to which factors contribute to the development of the disease and how they influence this development. If, at the end of the study, there is uncertainty about the cause of different disease exposures or drug effects, these should be clearly stated as unexplained factors. Conclusions or allusions to possible genetic differentiation should not be made, especially if other non-genetic factors have not been considered. Non-specific generic terms, even at the continental level, such as the term Asians or Africans, should be avoided as these encompass too large and genetically heterogeneous populations [127]. In addition, genetic studies too often equate a person's geographic assignment with geographic ancestry, which can lead to conceptual errors in many cases. Self-report of race is not directly equivalent to a person's ancestry. Therefore, individuals should not be assigned to a genetic ancestry group based on their race, even if they identify with it [128]. Because continental boundaries are largely politically determined, designations of origin are to some degree arbitrary and volatile over time. Thus, when genetic trait differences are identified between populations, it is important to emphasize that these represent probabilities and do not apply to every individual [129]. Hence, NASEM recommends avoiding typological thinking in relation to the OMB standards in human genetic research. The OMB standards were created to capture sociopolitical origins and not human biodiversity, yet they are used for biological classification in studies. They give a false picture of temporal continuity and homogeneity when used to describe genetic variation. Due to the imprecision in the description of genetic variability, the descriptions are prone to misunderstandings. The population geneticist Graham Coop criticizes that in studies that focus on genetic ancestry, factors such as kinship, environmental factors, social environment, and the place of data collection are mixed up. Scientists are therefore tempted to assume that identified “differences in genetic ancestry as a genetic cause of differences in phenotypic and health outcomes between groups” [130, p. 11].

Generally, one can observe that throughout the course of human evolution, there have always been mixtures and long-distance migrations. However, it is wrong to assume that initial homogeneous populations that would have mixed. There are no genetically homogeneous groups that were evolutionarily stable over a long period of time. When researchers understand ancestral groups as homogeneous groups, knowledge about genetic and environmental differences can be distorted [130]. Since all humans are related to each other in a large, complex family tree, it makes more sense to speak of genetic similarity [130]. Although the comparisons of genetic similarity are more complex in their application, they lead to a more accurate description of the genetically recorded background. The actual genetic variation between people depends on the proportion of the genome inherited from common ancestors and cannot be measured by recording a person's race [130].

## Summary

Since the emergence of the concept of race, there has been a constant renewal of the concept and a continuous racialization of human life and science. The concept of race was never a neutral or objective label to describe people but was created as a justification for the oppression of non-White people. Although the term has undergone a transformation and is now also understood as a sociocultural category, the problem of the meaning inscribed in the word remains [131, p. 271]. On the one hand, race is an empowering, identity-creating self-designation; on the other hand, race is used as a statistical category or for racial discrimination [132]. The classification of race is still primarily based on phenotypic characteristics, and the phenotypic understanding is maintained especially in pharmaceutical regulation, where race is translated as skin color or geographical ancestry. Pharmacogenetics has thus contributed significantly to further racialization. The transfer and application of racial categories to genetic drug studies reveals that the understanding of race is biological. In other words, the unquestioned inclusion of the social category of race in medical and pharmacogenetic studies results in race being understood as immutable and clearly defined biological phenomenon [16, p. 3; 132, 133, p. 488].

Simply addressing the definition of race can sensitize researchers to the discourse. Furthermore, researchers and clinicians must question their research and clinical algorithms that use race as a category regarding the implication and causal background for different disease prevalence [56, p. 880]. This can only be achieved by constantly reviewing one’s use of the category race. Therefore, we recommend that racism-critical education should already be implemented in medical and research training [134, p. 205].

In the future, different health outcomes should be assessed by considering the effects of racism on patient vulnerabiltiy. As Jennifer Tsai writes, “Rather than a risk factor that predicts disease or disability because of genetic susceptibility, race is better conceptualized as a risk marker—of vulnerability, bias or systemic disadvantage” [135]. Racism, not a person's race, is the cause of existing health inequalities. Various studies on race cannot be completely avoided, as they make a decisive contribution to the recording of racializing practices. Differences of a sociocultural nature, such as in health promotion, need further research to prevent further discrimination. The fundamental elimination of the category in studies would mean that one ignore the racializations used to label non-White people and affirm the assumption that all people have the same unrestricted opportunities to receive health care.

Both pharmacogeneticists and doctors occupy an important position in the language about race and racism. Their contributions, even when published in a supposedly protected scientific space, play an important role in the general perception [2]. The ambiguity of the term race and its attribution leads to the risk of participating in or perpetuating discriminatory practices of racism. Structural racism permeates all areas of life, including medicine, medical prescriptions, pharmacological regulations, and studies on personalized medicine. Medicine and medical action, such as medication prescription, are embedded in the socio-political history of humanity and science does not emerge in a neutral space that remains untouched by these norms and power structures. Not only does racism itself revitalize racist research in medicine, but the repeated practices in medicine to study it scientifically perpetuate it. When recording race in medical research, the aim should therefore always be to highlight and dismantle racism rather than to supposedly confirm racist theories or prove supposed biological differences. The aim of personalized medicine is to enable an individual therapy for each person, depending on their own genetic composition and individual characteristics, which in turn is an interplay of many factors, some of which coincide with the genetic composition of other people. However, race cannot be taken as a superordinate category that brings these factors together.

## Recommendations

In the following, the most important recommendations are briefly summarized to pay particular attention to the most important aspects.

- Establish interdisciplinary consensus on the appropriate use and utilization of data for human classification to help identify non-genetic and environmental factors contributing to disease prevalence, to avoid framing race as a biological determinant.
- Acknowledgements that differences in disease prevalence cannot be exclusively attributed to genetic predisposition but require a detection and utilization of specific risk alleles as a basis for universal screening rather than relying on racial proxies.
- Differentiate between individuals and populations—avoiding interpretation of individuals as representatives of racial or ethnic groups—while promoting the inclusion of diverse populations in biomedical research.
- Clearly state the rationale and methodology for using race categories, including how such data were collected, and distinguish between socio-cultural and biological factors, particularly when interpreting disease patterns and treatment responses.
- Avoid non-specific or overly broad terminology when describing populations or risk factors, and refrain from using OMB standards in genetic or pharmacogenetic research, as they were established only for sociopolitical classification.
- Incorporate racism-critical education into medical and scientific training.
- Investigate and account for racism as a structural risk factor affecting different health outcomes to always prioritize efforts to identify, analyze, and combat racism as a central objective in research utilizing race as a depictive category.

## Footnotes

1 The differences between two randomly selected individuals has since been revised to an estimate of 0.5 percent [4].

2 The one-drop rule is a legal principle of *racial* classification in the twentieth century in the USA. One drop of Black blood was sufficient to declare a person as Black. This served to develop a *racial* system and prevent miscegenation and equal opportunities.

3 Although in English usage there has been a reclaiming of the formerly purely biological concept of race and it is now used as a social category of self-attribution, in German it is still associated with a purely biological meaning. Despite the reclamation, it is problematic to use a social category in bioscientific studies, as it reinforces the link back to a biological basis.

4 Beta-blockers: Act primarily via beta-receptors on the heart. By competitively inhibiting these receptors, catecholamines are less effective and blood pressure is lowered. Consequently, they do not act via RAAS inhibition.
